# Vitamin Insufficiency After Esophagogastric Surgery and Targeted Supplementation: A Multicenter Prospective Intervention Study Named the VITAMIN Study

**DOI:** 10.3390/nu18183081

**Published:** 2026-09-20

**Authors:** Ariadne L. van der Velden, Sam S. I. Bartz, Kick J. E. M. Bluijssen, Thomas A. Vermeer, Nicola Lennartz, Kirsten A. Waanders, Charlène van der Zijden, Sander J. M. van Hootegem, Lotte Blonk, Bjorn Winkens, Evert-Jan G. Boerma, Jan H. M. B. Stoot, Eric H. J. Belgers, Suzanne S. Gisbertz, Bas P. L. Wijnhoven, Mathie P. G. Leers, Meindert N. Sosef, Guy H. E. J. Vijgen

**Affiliations:** 1 Department of Surgery, Zuyderland Medical Center, Dr. H. Van Der Hoffplein 1, 6162 BG Sittard-Geleen, The Netherlands; 2 Department of Surgery, Erasmus Medical Center, r. Molewaterplein 40, 3015 GD Rotterdam, The Netherlands; 3 Department of Surgery, Amsterdam University Medical Center, Location Vrije Universiteit Amsterdam, De Boelelaan 1117, 1081 HV Amsterdam, The Netherlands; 4 Department of Methodology and Statistics, Maastricht University Medical Center+, Debyelaan 25, 6229 HX Maastricht, The Netherlands; 5 Care and Public Health Research Institute (CAPHRI), Maastricht University, Universiteitssingel 50, 6229 ER Maastricht, The Netherlands; 6 Department of Clinical Chemistry and Hematology, Zuyderland Medical Center, Dr. H. Van Der Hoffplein 1, 6162 BG Sittard-Geleen, The Netherlands

**Keywords:** esophagectomy, gastrectomy, malnutrion, micronutrient deficiency

## Abstract

Background/aim: Guidelines on screening for pre or postoperative micronutrients are currently lacking for patients diagnosed with gastric or esophageal cancer. this study aimed to identify micronutrient deficiencies after surgery for esophagogastric neoplasms and to evaluate changes in micronutrient status during targeted supplementation. Methods: This was a non-randomized prospective intervention trial conducted in The Netherlands between December 2021 and December 2024. Patients who underwent esophagectomy or (sub)total gastrectomy were included. At baseline (T0), blood measurement prior to supplementation was performed. All measurements were repeated after six (T1) and 12 months (T2) of supplementation. Both groups received Calcium Soft Chew D3 and a multivitamin supplement depending on the performed surgery (i.e., Multi-E or Multi-G). Results: In total, 107 patients were included (*n* = 76 esophagectomy group). At T0, at least one micronutrient deficiency was present in 83.9% of patients after gastrectomy (median 30.1 [IQR: 19.4–148.4] weeks after surgery) and in 67.1% of patients after esophagectomy (median 36.6 [IQR: 14.4–108.3] weeks after surgery). The four most common deficiencies at baseline were vitamin D (36.8%), iron (32.7%), zinc (22.8%), and ferritin (20.8%). For vitamin D and iron an increase in concentrations during follow-up was seen (*p* < 0.002), except for zinc in the esophagectomy group (*p* < 0.001). Conclusion: During targeted supplementation, the prevalence of several micronutrient deficiencies decreased and several micronutrient concentrations increased. Further prospective randomized studies are needed to confirm these findings.

## 1. Introduction

Esophageal cancer (EC) and gastric cancer (GC) were among the top six causes of cancer-related death worldwide in 2022 [1]. Both diseases can result in weight loss and malnutrition, which has been reported in 42 to 80% of patients [2,3,4,5]. Currently, surgery is part of multidisciplinary treatment with curative intent for patients diagnosed with EC or GC.

Due to surgical resection of the esophagus and/or stomach and the method of reconstruction of the gastrointestinal tract, the in- and uptake of nutrients can be compromised [6,7]. Other postoperative physiological changes including digestive hormone alterations and vagal denervation can impair digestive enzyme stimulation and thereby lead to exocrine pancreatic insufficiency. In addition, patients often experience inadequate oral intake for months to years after surgery and are required to modify their eating patterns [6]. Consequently, patients experience maldigestion, malabsorption and weight loss, which affect quality of life and lead to malnutrition [8,9]. Postoperative malnutrition is commonly present, as up to 87% of patients diagnosed with upper gastrointestinal cancers have malnutrition after treatment with curative intent [10].

Malnutrition can result in micronutrient deficiencies [7]. The prevalence of minimal one micronutrient deficiency in patients who have undergone esophagogastric surgery for cancer is 60–78% in literature [11,12,13,14]. Given the altered anatomy and physiology after surgery, uptake via a normal dietary intake may however be insufficient, and additional supplementation is possibly necessary [11,12]. Nonetheless, international guidelines recommending monitoring and treatment of micronutrient deficiencies in patients who underwent esophagogastric surgery due to malignancy are currently lacking. Hence, the primary aim of this study was to identify micronutrient deficiencies after surgery for esophageal and gastric cancer, and secondary to study changes in concentration levels during routine supplementation. We hypothesized that most patients had micronutrient deficiencies after surgery and that targeted supplementation would resolve most of the diagnosed deficiencies.

## 2. Materials and Methods

### 2.1. Study Population

The VITAMIN (VITAMin Insufficiency in esophagogastric Neoplasms) study was a non-randomized Dutch multicenter prospective intervention study conducted between December 2021 and December 2024. The study was performed in the Zuyderland Medical Center (MC), Erasmus MC and the Amsterdam University MC (UMC). This study was initially approved by the Ethics Committee of Zuyderland Medical Center (METCZ20210146).

Patients ≥ 18 years of age who underwent curative esophagectomy or gastrectomy (subtotal or total) for malignancy with no signs of disease recurrence between 2011 and 2023 were eligible to participate. Patients were identified at the participating centers and were approached for study participation by the research team, either by telephone or during an outpatient follow-up visit. As no systematically screening/eligible-numbers were thoroughly recorded, a flow diagram was not provided. No particular inclusion criteria were present regarding the surgical reconstructions, except for wedge resection, which was an exclusion criterion. The minimal interval time between surgery and inclusion was six weeks, and no maximum time interval was selected. Exclusion criteria besides wedge resection were recurrent malignant disease, metastases, ongoing treatment with chemotherapy and incapability of oral intake during inclusion and study period. This study was conducted and reported in accordance with the Transparent Reporting of Evaluations with Nonrandomized Designs (TREND) statement [15]. More detailed information about the study methods can be found in the published study protocol [16].

### 2.2. Study Algorithm

**Figure 1:**
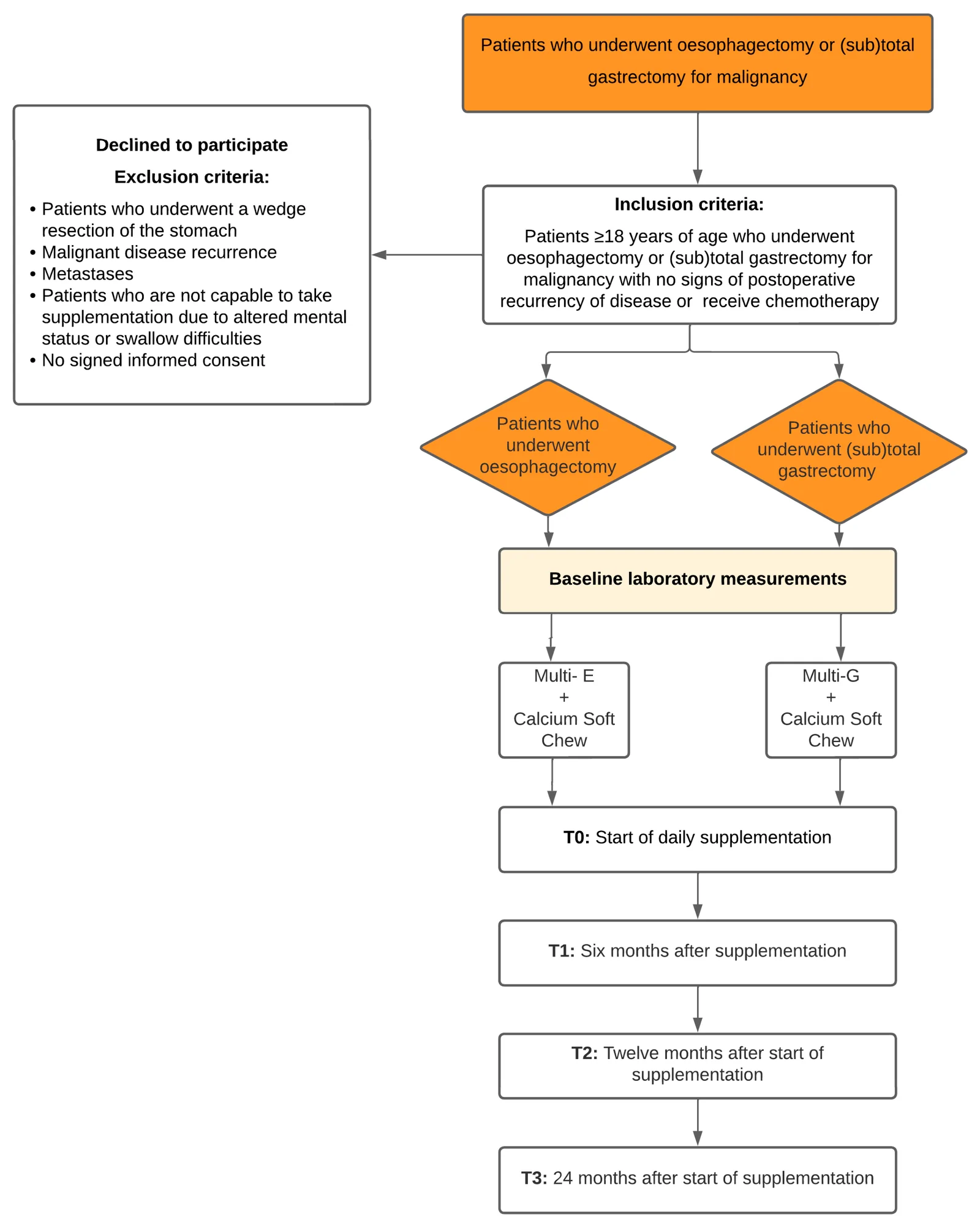
Overview of the study design. Patients are divided in the study group based on the surgery they have undergone. Patients who underwent esophagectomy received multi-E and patients who underwent gastrectomy received Multi-G. Both groups received Calcium Soft Chew simultaneously with the multivitamin, and underwent blood analyses on T0, T1, T2, and T3.

Patients were divided in intervention groups according to the surgery performed (i.e., (sub)total gastrectomy or esophagectomy group). No additional procedures, such as matching or stratification were applied. The two study groups were not considered comparable at baseline, as both patient groups had distinct underlying diseases and underwent different surgical procedures associated with different risks of developing micronutrient deficiencies. Hence, between-group comparisons were not performed. No blinding was applied. All included patients underwent blood withdrawal at baseline (T0), 6 months after baseline (T1), 12 months after baseline (T2), and 24 months after baseline (T3) (Figure 1). Before the start of the study, a sample size of 124 patients per surgical group was calculated based on a Cohen’s d = 0.4, a two-sided alpha of 0.05, a statistical power of 80%, and an anticipated drop-out rate of 20%. This sample size calculation was not derived from a specific micronutrient outcome or previous study, as comparable intervention data were not available at the time of study design.

### 2.3. Blood Analysis

Venous blood sampling was collected to analyze different nutrient states at all time points. The first venous blood sampling was collected postoperatively before supplementation. The analyzed parameters included vitamins (A, B1, B6, B12, D, E, folate), minerals (serum iron, transferrin, ferritin, transferrin saturation, zinc, magnesium, phosphate), and parathyroid hormone (PTH). Deficiencies were classified according to guidelines of the laboratory of all three centers. The thresholds for deficiency per center can be found in the Supplementary Material (Supplementary Table S1).

### 2.4. Multivitamin Supplement

GIKAVI BV (Rotterdam, the Netherlands) developed three study supplements specifically for patients who had undergone esophagectomy or gastrectomy [17]. The formulation was based on published evidence regarding postoperative nutrient deficiencies in these patient groups and on multivitamin supplements commonly prescribed after bariatric surgery. The ingredients per supplement can be found in Supplementary Table S2. Patients who underwent esophagectomy received Multi-E and patients who underwent gastrectomy received Multi-G. Both groups received the same Calcium Soft Chew D3 prescription. The multivitamin supplement was administered once daily and Calcium Soft Chew D3 twice daily. Patients who took vitamin supplements in any form at baseline were asked to stop their own supplementation. At baseline, patients received supplements for 180 days. After 6 months, patients received supplements for another 180 days. After 360 days of supplementation, patients received supplements for 360 days. The supplements were provided on the day of the hospital visit for blood analysis at the participating center. Adherence was intended to be assessed during each follow-up visit by evaluating the remaining study supplements returned by patients. However, adherence data were incomplete because the remaining supplements were overall not consistently available for assessment at each visit.

### 2.5. Study Parameters/Endpoint

The primary outcome in this study is the proportion of patients with deficiency (yes/no) of vitamins and minerals measured in the nutrient blood levels below the specified threshold used per center at baseline and whether these were resolved during follow-up. The secondary parameter was the change of vitamin concentrations over time, measured separately for both surgical groups.

### 2.6. Safety and Stopping Rules

Disease recurrence, treatment with chemotherapy or severe hypervitaminosis, defined as a blood concentration above the upper limit of the reference range used by the participating center, except for vitamin B12, resulted in exclusion at baseline.

### 2.7. Statistical Analysis

Continuous variables were presented as mean (±SD) for normally distributed data or as median with 1st interquartile and 3rd interquartile [IQR] for non-normally distributed data. Categorical variables were expressed as absolute numbers and percentages (*n*, %). Continuous variables were assessed for normality. For non-normally distributed continuous variables, differences between two subgroups were assessed using the Mann–Whitney U test. The changes in deficiencies (yes/no) over time (T0 vs. T2) were analyzed using McNemar’s test for patients who completed follow-up until T2. Micronutrient changes over time were analyzed using linear mixed-effects models (LMM) with time as a categorical fixed effect and a random intercept for each participant to account for within-subject correlation across repeated measurements of micronutrient levels. Values of T1 and T2 were compared to baseline measurements. Missing values were not imputed, as a likelihood-based approach was used, allowing all available observed data to be included in the analysis. Due to loss of follow-up and early termination of the study resulting from the expiration of the supplements and slower accrual rate than initially expected, T3 data were excluded from the analyses to retain statistical power. Additionally, complete-case LMM analysis were performed among patients who completed follow-up until T2, and had no missing micronutrient concentration data at T0, T1, or T2.

Only laboratory values measured with similar methods were included for LMM analyses, and therefore zinc measured at Erasmus MC, and vitamin K for both Erasmus and Amsterdam UMC, were excluded. Residual normality of all time points was evaluated to verify model adequacy. If the normality assumption was not met, log-transformation was performed. Consecutively, back transformation was performed to obtain estimated geometrical means with corresponding 95% confidence intervals (CIs). In addition, the ratios of geometric means (T1 vs. T0 and T2 vs. T0) are presented with corresponding *p*-values. Normally distributed laboratory values were presented as estimated marginal means (EMM) with standard error (SE). Additionally, differences between time points were shown as EMM differences with 95% CIs and *p*-values.

Missing data of variables of interest were noted, and the number of available values for all variables of interest was reported in percentages. Sensitivity analyses were performed to compare excluded patients with included patients per follow-up moment to assess potential selection bias. The results of the sensitivity analyses are in Supplementary Tables S3–S6.

All data was captured from the electronic patient file and collected in Research manager (Deventer, The Netherlands), and analyzed using IBM Statistical Package for Social Sciences (SPSS) version 29 (Armonk, NY, USA). A two-sided *p*-value ≤0.05 was considered statistically significant. However, given the exploratory nature of the LMM analyses and the number of micronutrients assessed, a two-sided significance level of α = 0.01 was applied.

## 3. Results

### 3.1. Baseline Characteristics

In total, 107 patients met the inclusion criteria. In this study population, 85 patients were included from Zuyderland MC (*n* = 59 esophagectomy), 7 from AUMC (*n* = 5 esophagectomy), and 15 from Erasmus MC (*n* = 12 esophagectomy). Baseline characteristics are shown in Table 1. In the (sub)total gastrectomy group, 31 patients were included (67.7% male, median age = 69 [IQR: 59–74]), and 76 patients were included in the esophagectomy group (85.5% male; median age 69 [IQR: 61–73]). In the gastrectomy group, 12 patients underwent subtotal gastrectomy and 19 total gastrectomy. The median time from surgery to baseline measurement was 30.1 [IQR: 19.4–148.4] weeks for the gastrectomy group, and 36.6 [IQR: 14.4–108.3] weeks for the esophagectomy group. Use of vitamin supplements was mostly seen in the gastrectomy group (55.2%). Prior to study participation, 46.7% of the patients in the gastrectomy group received vitamin B12 injections; all of these patients had undergone total gastrectomy.

During complete follow-up, 50 patients were excluded. Most common reasons were hypervitaminosis at baseline (*n* = 10) and disease progression (*n* = 21), of which fifteen patients had recurrence and six patients passed away. Additionally, sixteen patients stopped due to possible side effects. The following effects were reported after start of supplementation: obstipation (*n* = 7), nausea (*n* = 7), diarrhea (*n* = 1), pruritus (*n* = 1). Three patients withdrew due to dissatisfaction with the supplement. Per-time-point sensitivity analyses showed no statistically significant differences between patients who reached T1 and patients who were excluded during follow-up until T1 for both the esophagectomy and gastrectomy group. From T1 to T2 significant differences were only found for age (*p* = 0.004) in the gastrectomy group, and in ASA classification for the esophagectomy group (*p* = 0.026) (Supplementary Tables S3–S6).

### 3.2. Micronutrient Deficiencies at Baseline

At baseline, 72.0% of the total population (67.1% in the esophagectomy group; 83.9% in the gastrectomy group) showed at least one micronutrient deficiency. The four most common deficiencies at baseline were vitamin D (36.8%), iron (32.7%), zinc (22.8%), and ferritin (20.8%) (Table 2). Within the gastrectomy group, the prevalence of at least one deficiency at baseline was 66.7% among patients who underwent subtotal gastrectomy and 94.7% among those who underwent total gastrectomy. In the (sub)total gastrectomy group, median time since surgery was 54.9 [IQR: 29.6–376.6] weeks among patients without a micronutrient deficiency at baseline and 27.5 [IQR: 19.4–104.1] weeks among patients with at least one deficiency (*p* = 0.257). When stratified by supplement use prior to inclusion, at least one micronutrient deficiency was present in 84.6% of patients without prior supplementation and 81.3% of patients with prior supplementation in the gastrectomy group (*p* = 1.000). For the gastrectomy group, median time since surgery was 30.1 [IQR: 14.9–131.7] weeks in patients without prior supplementation and 47.1 [IQR: 20.0–152.2] weeks in patients with prior supplementation (*p* = 0.682).

In the esophagectomy group, median time since surgery was 33.1 [IQR: 14.9–110.8] weeks among patients without a micronutrient deficiency at baseline, and 41.3 [IQR: 13.7–103.9] weeks among patients with at least one deficiency (*p* = 0.894). The prevalence of at least one micronutrient deficiency among patients who had no prior supplementation versus patients with prior supplementation were 75.6% and 58.3%, respectively (*p* = 0.174) (Supplementary Table S8). Nonetheless, a significantly higher prevalence of Vitamin D was found for the group that did not use prior supplements compared to the group that used prior supplements (45.5% versus 12.5%, respectively; *p*-value = 0.007).

Median time since surgery was 22.7 [IQR: 11.5–92.0] weeks among patients not using vitamin supplements and 90.2 [IQR: 28.3–127.6] weeks among patients using vitamin supplements. A statistically significant difference in time since surgery was observed between patients with and without prior supplementation (*p* = 0.013).

### 3.3. Changes in Micronutrient Deficiencies over Time

The prevalence of micronutrient deficiencies for complete case analyses at T0 and T2 can be found in Table 3. Among patients who underwent gastrectomy, the prevalence of vitamin D and ferritin decreased significantly from T0 to T2 (*p* ≤ 0.031). No statistically significant changes were observed for the other micronutrient deficiencies.

In the esophagectomy group, significant decreases in prevalence between T0 and T2 were observed for vitamin D, zinc, and iron (*p* ≤ 0.039) (Table 4). In contrast, phosphate deficiency prevalence increased significantly from 2.4% at T0 to 19.0% at T2 (*p* = 0.016).

### 3.4. Micronutrient Concentrations over Time

In the gastrectomy group, several micronutrient concentrations changed significantly over time (Table 5). Concentrations of vitamin B1 and vitamin A increased significantly at both follow-up moments compared with baseline (both *p* < 0.001). For the log-transformed outcomes, significant time effects were shown for folate, vitamin B12, vitamin D, ferritin, iron, and zinc (all *p* < 0.001). Compared to baseline, folate, vitamin B12 and vitamin D concentrations increased substantially at T2 with ratios of 2.23, 1.75, and 2.47, respectively. Zinc concentrations also increased significantly over time, reaching a ratio of 1.33 at T2 (*p* < 0.001). Conversely, PTH concentrations decreased significantly over time (*p* < 0.001), with lower levels at both T1 and T2 compared to baseline (both *p* < 0.001).

In the esophagectomy group, several micronutrient concentrations also significantly changed over time (Table 6). Significant overall effects of time were observed for vitamin A (*p* = 0.005), calcium (*p* = 0.006), and iron (*p =* 0.002) concentrations. For the log-transformed outcomes, significant time effects were observed for vitamin B1 (*p* < 0.001), vitamin B6 (*p* = 0.005), folate (*p* < 0.001), vitamin B12 (*p* < 0.001), vitamin D (*p* < 0.001), and zinc (*p* < 0.001) concentrations. Concentrations of folate, vitamin B12, and vitamin D increased substantially, with ratios at T2 of 2.68, 1.65, and 2.24, respectively. Zinc concentrations decreased over time as the ratio at T2 was 0.86 (*p* < 0.001).

Complete-case sensitivity analyses for all micronutrient concentrations showed comparable overall findings in both study groups (Supplementary Tables S9 and S10).

## 4. Discussion

This non-randomized study aimed to explore micronutrient deficiencies after esophagogastric surgery and to assess whether targeted vitamin supplementation could correct deficiencies in patients with gastric or esophageal cancer. We found that at baseline, 72.0% of included patients had at least one micronutrient deficiency. The most prominent deficiencies were vitamin D, iron, zinc and ferritin. In the gastrectomy group, significant changes in micronutrient concentrations at T2 were observed for vitamin B1, vitamin A, folate, vitamin B12, vitamin D, ferritin, iron, and zinc while PTH concentrations decreased. Regarding changes in deficiency status, the prevalence of vitamin D and ferritin deficiencies was significantly lower in complete-case analysis. In the esophagectomy group, significant changes in concentrations at T2 compared to baseline were observed for vitamin A, calcium, iron, vitamin B1, vitamin B6, folate, vitamin B12, vitamin D, whereas zinc concentrations decreased. Vitamin D, iron and zinc deficiency prevalence decreased significantly in complete-case analysis, although phosphate deficiency was significantly more prevalent at T2.

Currently, no comprehensive guideline exists for postoperative monitoring of different micronutrients after esophagogastric surgery. By contrast in bariatric surgery, in which similar anatomical alterations are performed to those undergoing esophagectomy or (sub)total gastrectomy, regular biochemical monitoring, combined with procedure-specific vitamin supplementation is implemented as standard of care [18]. For patients undergoing gastrectomy intramuscular vitamin B12 supplementation is recommended to prevent deficiency, and it is advised to yearly monitor iron concentrations and treat iron deficiency if necessary. For patients with esophageal cancer, vitamin B12 supplementation after surgery is not generally recommended. It is however advised to preoperatively assess micronutrient deficiencies as baseline measurement, and to assess micronutrient levels (i.e., vitamin D, vitamin B12, iron and zinc) six months after surgery [19]. However, clear recommendations regarding broader postoperative micronutrient monitoring and targeted supplementation remain limited [20].

Several studies have retrospectively assessed micronutrient status in patients undergoing esophagogastric surgery due to malignancy, and have demonstrated that micronutrient deficiencies are common in this population [11,13,14,21,22]. In a retrospective study, Blonk et al. reported that most prevalent deficiencies after esophagectomy and gastrectomy were iron (35.8% and 33.3%), vitamin D (32.9% and 52.4%), zinc (19.7% and 27.6%), and ferritin (10.8% and 16.7%), respectively [13]. Similarly, Jansen et al. found that 78.3% of patients had at least one micronutrient deficiency after esophagectomy, most commonly involving vitamin D (50.6%), iron (42.2%), folate (28.8%), and vitamin B12 (18.3%) [14]. In our study, we found a lower prevalence, as 67.1% of the patients who underwent esophagectomy had at least one micronutrient deficiency. Nonetheless, vitamin D and iron were also among the most prevalent deficiencies in this group, although folate and vitamin B12 deficiencies were less common. A prospective study performed by Oberhoff et al. assessed nutritional status before and six months after esophagectomy and reported postoperative decreases in vitamin B12 and ferritin concentrations [23]. These findings illustrate that micronutrient concentrations may decline during postoperative follow-up and underline the potential importance of continued micronutrient monitoring and supplementation when indicated.

A study by Veeralakshmanan investigated the effect of a standard multivitamin supplement with additional intravenous iron and/or vitamin B12 injections when necessary in patients who had undergone esophagogastric surgery [11]. Significant improvements in concentrations were observed for ferritin, folate, vitamin D, and vitamin B12 concentrations, although patients after esophagectomy and gastrectomy were analyzed as a combined cohort. In the present study, folate, vitamin D, and vitamin B12 concentrations increased in both surgical groups, whereas ferritin concentrations solely increased in the gastrectomy group. Importantly, 46.7% of patients in the gastrectomy group had received vitamin B12 injections before study participation, and one patient required an additional vitamin B12 injection during the intervention period. These factors should be considered when interpreting the vitamin B12 findings. Furthermore, the supplementation strategies differed, as the supplements evaluated in the present study were specifically formulated according to the anticipated micronutrient requirements after esophagectomy or gastrectomy, whereas Veeralakshmanan et al. used a standard multivitamin supplemented with additional treatment when indicated.

Micronutrient concentrations after esophagogastric surgery have also been compared with healthy populations. A meta-analysis by Finze et al. evaluated postoperative micronutrient status with reference values from healthy populations [22]. The pooled mean vitamin D concentration among 293 patients was 27.51 ng/mL, (reference range: 30–100 ng/mL). Mean vitamin B12 concentrations were also lower in patients after esophagogastric surgery than in the healthy population (536.59 versus 600 ng/mL; *p* < 0.001), as were mean calcium concentrations (9.16 versus 9.45 mg/dL; *p* < 0.001). In the present study, 36.8% of the patients had a vitamin D deficiency, but lower vitamin B12 deficiency at baseline (3.8%), as well as calcium deficiency (8.5%) numbers were found. This discrepancy can possibly be explained by vitamin use prior to inclusion as 40.8% (40 out of 98 patients) of the study population used vitamin supplements in any form prior to inclusion. Most importantly, vitamin B12 injections were used by 46.7% of the gastrectomy population. Therefore, the relatively low prevalence of vitamin B12 deficiency at baseline should be interpreted in the context of previous vitamin B12 treatment, and treatment-naïve postoperative baseline is mostly missing in our cohort study. Another explanation for differences in outcomes is the differences in follow-up duration. The median time from surgery to baseline measurements in our study was 36.6 [IQR: 14.4–108.3] weeks in the esophagectomy group, and 30.1 [IQR: 19.4–148.4] weeks in the gastrectomy group. In contrast, the studies included in the meta-analysis by Finze et al. assessed micronutrient levels over a broader postoperative interval in a larger sample size, ranging from 3 months up to 10 years after surgery [22]. Such heterogeneity in follow-up timing may influence the reported prevalence of micronutrient deficiencies. Micronutrient levels may change over time because of nutritional intake, supplementation and postoperative adaptation.

Micronutrient deficiencies are also present in the general population. In a Dutch study including 348 healthy participants (mean age 44.6 ± 11.1 years), deficiencies in vitamin B6, vitamin B12, vitamin D and iron were observed in 0.9%, 0.6%, 1.1% and 4.0% of individuals, respectively, compared to 3.0%, 3.8%, 36.8% and 32.7% in our cohort [24]. In another Dutch study, vitamin D deficiency was observed in 7% of women and 13% of men aged 50–69, and in 26% of men and 13% of women aged ≥ 70 years [25]. In our cohort, 36.8% of the study population was deficient for vitamin D. These findings suggest that clinically relevant deficiencies are more prevalent in patients with gastroesophageal cancer than in the general population. However, because micronutrient concentrations were not measured before surgery, it remains unknown whether some patients were already deficient preoperatively, which is a limitation of the study design.

Other study limitations should also be considered. Prior to the study, a power calculation was performed which suggested an optimal number of 248 patients who underwent esophagectomy (*n* = 124) and gastrectomy (*n* = 124) had to be included to show an effect. Nonetheless, we only included 76 patients in the esophagectomy group and 31 patients in the gastrectomy group which increases the risk of a type II error. Also, the high loss-to-follow-up rate may have introduced attrition bias, as loss to follow-up was mainly related to disease recurrence, death, or side effects. Thus, the patients remaining in follow-up may not be fully representative of the broader population undergoing gastrectomy or esophagectomy for malignancy. Moreover, no untreated control group was included in the study. Although longitudinal changes in micronutrient status were observed during supplementation, these changes cannot be fully attributed to the use of targeted supplements, as other clinical, dietary or postoperative factors may have contributed. An untreated comparison group was considered inappropriate, as withholding supplementation from patients at known risk of micronutrient deficiencies, particularly vitamin B12 deficiency after gastrectomy, could potentially result in deficiencies and symptoms. Furthermore, patients undergoing subtotal and total gastrectomy were combined into a single gastrectomy cohort. This approach was chosen to preserve statistical power, as stratification by type of gastrectomy would have resulted in smaller subgroup sizes. Consequently, stratified analyses of longitudinal changes in micronutrient concentrations according to prior supplementation status were not performed because of the limited sample size and further attrition during follow-up. Similarly, the influence of the interval between surgery and baseline measurements on longitudinal changes in micronutrient concentrations was not assessed. Additionally, the original sample size calculation was based on a between-group effect, whereas the two groups were not considered directly comparable in the present study. For this reason, the applicability of the original sample size calculation to the current analyses is uncertain. Nonetheless, to our knowledge, this is the first prospective study evaluating targeted micronutrient supplementation in patients who underwent esophagectomy or gastrectomy. Moreover, the prospective design enhances the validity of the findings. Another limitation hampering statistical power is the high rate of loss to follow-up, which is partly related to poor prognosis of esophagogastric cancer. To increase and retain power, it was therefore decided to include patients who underwent surgery from 2011 until 2023, and to perform analyses until T2, instead of T3. For the latter, sensitivity analyses were performed to assess differences between the included and the excluded patients during T1 and T2 and only minor differences were found in both study groups. However, the interval between surgery and study enrollment varied between patients. In additional analyses, no significant differences in time since surgery between patients with and without a deficiency were observed in either intervention group. Nonetheless, because patients entered the study at different postoperative time points, the potential relationship between time since surgery and micronutrient status could not be established in our study. Moreover, the exclusion of patients with recurrent or metastatic disease, ongoing chemotherapy, or inability to take oral supplementation may have introduced selection bias. Adherence could also not be thoroughly assessed, as these data were incomplete for the majority of patients. Our findings should therefore be interpreted as exploratory with respect to postoperative timing and should not be considered representative of micronutrient status during a specific postoperative period.

Lastly, deficiency status was determined using center-specific laboratory reference values. Although this may have introduced some heterogeneity in the classification of deficiencies, these reference ranges were specific to the laboratory methods and assays used per participating center. Therefore, applying a single uniform cut-off across centers may not necessarily improve comparability and could potentially result in misclassification. This should be considered when interpreting the reported prevalence of micronutrient deficiencies. Nevertheless, the potential impact of between-center variation may have been limited in the present study, as the majority of patients were included at a single center.

Future research should evaluate postoperative micronutrient supplementation strategies in larger multicenter cohorts, with a shorter interval between surgery and start of monitoring and longer follow-up to prevent both deficiencies and excessive micronutrient concentrations. Moreover, comparison with standard-of-care is necessary to further assess the impact of targeted supplementation. In addition to biochemical outcomes, future studies should assess whether abnormal micronutrient concentrations are also associated with symptoms or other clinically meaningful outcomes, and whether biochemical deficiencies reflect functional deficiencies or deficiencies at tissue-level. As clinical symptoms were not systematically assessed in the present study, it remains unclear to what extent the observed lower concentrations translate into clinically relevant consequences.

## 5. Conclusions

Micronutrient deficiencies are present in patients undergoing esophagogastric surgery. During targeted supplementation, the prevalence of several micronutrient deficiencies decreased and several micronutrient concentrations increased. Further prospective randomized studies are needed to confirm these findings.

## Figures and Tables

**Table 1:** Baseline characteristics for both groups.

Baseline Characteristics *N* = 107	Gastrectomy Group *n* = 31	Esophagectomy Group *n* = 76
Male (*n*, %)	21 (67.7)	65 (85.5)
Median age [IQR]	69 [59–74]	69 [61–73]
Median BMI in kg/m^2^ [IQR]	22.0 [20.0–24.0]	24.0 [22.0–27.0]
Smoking (*n*, %)	3 (9.7)	5 (6.6)
Diabetes Mellitus	1/30 (3.3)	5 (6.6)
Use of vitamin supplements	16/29 (55.2)	24/69 (34.8)
Vitamin B12 injections	14/30 (46.7)	4/75 (5.3)
ASA-score (*n*, %)	*n* = 31	*n* = 75
1	7 (22.6)	17 (22.4)
2	20 (64.5)	48 (63.2)
3	4 (12.9)	10 (13.2)
Median time in weeks from surgery to baseline measurement [IQR]	30.1 [19.4–148.4]	36.6 [14.4–108.3]
ypTNM staging (*n*, %)		
ypT-stage	*n* = 31	*n* = 76
ypT0	2 (6.5)	9 (11.8)
ypT1a	1 (3.2)	4 (5.3)
ypT1b	5 (16.1)	10 (13.2)
ypT2	6 (19.4)	15 (19.7)
ypT3	15 (48.4)	29 (38.2)
ypT4a	1 (3.2)	-
ypTx	1 (3.2)	9 (11.8)
ypN-stage	*n* = 31	*n* = 76
ypN0	18 (58.1)	49 (64.5)
ypN1	8 (25.8)	18 (23.7)
ypN2	3 (9.7)	7 (9.2)
ypN3	2 (6.5)	2 (2.6)

In the gastrectomy group *n* = 19 underwent total gastrectomy. ASA = American Society for Anesthesiologists, BMI = body mass index, ypTNM = tumor node metastasis staging after neoadjuvant therapy and surgery.

**Table 2:** Micronutrient deficiencies at T0.

Micronutrient Deficiencies at Baseline	Gastrectomy Group *n* = 28–31, *n* (%)	Esophagectomy Group *n* = 68–76, *n* (%)	Total *n* = 96–107, *n* (%)
At least one deficiency	26/31 (83.9)	51/76 (67.1)	77/107 (72.0)
Vitamin A	0/28 (0.0)	1/68 (1.5)	1/96 (1.0)
Vitamin B1	1/30 (3.3)	1/71 (1.4)	2/101 (2.0)
Vitamin B6	2/30 (6.7)	1/70 (1.4)	3/100 (3.0)
Folate	1/30 (3.3)	5/75 (6.7)	6/105 (5.7)
Vitamin B12	2/31 (6.5)	2/75 (2.7)	4/106 (3.8)
Vitamin D	16/31 (51.6)	23/75 (30.7)	39/106 (36.8)
Vitamin E	0/28 (0.0)	0/68 (0.0)	0/96 (0.0)
Calcium	4/31 (12.9)	5/75 (6.7)	9/106 (8.5)
Ferritin	12/30 (40.0)	10/76 (13.2)	22/106 (20.8)
Iron	12/31 (38.7)	23/76 (30.3)	35/107 (32.7)
Magnesium	0/31 (0.0)	2/75 (2.7)	2/106 (1.9)
Phosphate	0/31 (0.0)	1/75 (1.3)	1/106 (0.9)
Zinc	9/29 (31.0)	14/72 (19.4)	23/101 (22.8)

**Table 3:** Micronutrient deficiencies at T0 and T2 in the gastrectomy group as complete-case analysis.

Micronutrient Deficiency	T0 (*n*, %)	T2 (*n*, %)	Difference T0–T2, Percentage Points (95% CI)	*p*-Value
Vitamin A	0/15 (0.0)	0/15 (0.0)	0.0 (−20.4 to 20.4)	—
Vitamin B1	0/17 (0.0)	0/17 (0.0)	0.0 (−18.4 to 18.4)	—
Vitamin B6	0/17 (0.0)	0/17 (0.0)	0.0 (−18.4 to 18.4)	—
Folate	1/16 (6.2)	0/16 (0.0)	6.3 (−13.8 to 28.3)	1.000
Vitamin B12	0/17 (0.0)	0/17 (0.0)	0.0 (−18.4 to 18.4)	—
Vitamin D	10/17 (58.8)	0/17 (0.0)	58.8 (29.5 to 78.4)	0.002 *
Vitamin E	0/15 (0.0)	0/15 (0.0)	0.0 (−20.4 to 20.4)	—
Calcium	3/17 (17.6)	0/17 (0.0)	17.6 (−4.1 to 41.0)	0.250
Ferritin	8/17 (47.1)	2/17 (11.8)	35.3 (7.8 to 57.2)	0.031 *
Iron	5/17 (29.4)	1/17 (5.9)	23.5 (−1.5 to 47.2)	0.125
Magnesium	0/17 (0.0)	0/17 (0.0)	0.0 (−18.4 to 18.4)	—
Phosphate	0/17 (0.0)	1/17 (5.9)	−5.9 (−27.0 to 13.2)	1.000
Zinc	5/15 (33.3)	1/15 (6.7)	26.7 (−5.3 to 53.2)	0.219

* Statistical significance was set at α = 0.05. T0 = baseline, T2 = twelve months after supplementation.

**Table 4:** Micronutrient deficiencies at T0 and T2 in the esophagectomy group as complete-case analysis.

Micronutrient Deficiency	T0 (*n*, %)	T2 (*n*, %)	Difference T0–T2, Percentage Points (95% CI)	*p*-Value
Vitamin A	1/35 (2.9)	0/35 (0.0)	2.9 (−7.3 to 14.5)	1.000
Vitamin B1	1/40 (2.5)	0/40 (0.0)	2.5 (−6.5 to 12.9)	1.000
Vitamin B6	0/40 (0.0)	0/40 (0.0)	0.0 (−8.8 to 8.8)	-
Folate	2/39 (5.1)	0/39 (0.0)	5.1 (−4.6 to 16.9)	0.500
Vitamin B12	2/42 (4.8)	0/42 (0.0)	4.8 (−4.3 to 15.8)	0.500
Vitamin D	16/42 (38.1)	0/42 (0.0)	38.1 (22.5 to 53.2)	<0.001 *
Vitamin E	0/35 (0.0)	0/35 (0.0)	0.0 (−9.9 to 9.9)	—
Calcium	5/42 (11.9)	0/42 (0.0)	11.9 (1.2 to 25.0)	0.063
Ferritin	6/43 (14.0)	1/43 (2.3)	11.6 (−1.0 to 25.2)	0.125
Iron	17/43 (39.5)	8/43 (18.6)	20.9 (3.3 to 37.0)	0.035 *
Magnesium	1/42 (2.4)	1/42 (2.4)	0.0 (−10.2 to 10.2)	1.000
Phosphate	1/42 (2.4)	8/42 (19.0)	−16.7 (−30.8 to −4.1)	0.016 *
Zinc	9/35 (25.7)	2/35 (5.7)	20.0 (3.9 to 36.3)	0.039 *

* Statistical significance was set at α = 0.05. T0 = Baseline, T2 = Twelve months after supplementation.

**Table 5:** Micronutrient concentrations over time for the gastrectomy group.

Micronutrient	Follow-Up Moment	Linear Mixed Model			
		Estimated Marginal Mean (±SE)	Mean Difference (95%CI)	*p*-Value Mean Difference	*p*-Value Overall Time
Vitamin B1	T0	144.9 (8.1)	Ref.	Ref.	<0.001 *
	T1	191.5 (9.2)	46.6 (29.9–63.4)	<0.001 *	-
	T2	193.2 (9.7)	48.3 (30.6–65.9)	<0.001 *	-
Vitamin A	T0	1.7 (0.12)	Ref.	Ref.	<0.001 *
	T1	2.1 (0.13)	0.38 (0.18–0.59)	<0.001 *	-
	T2	2.2 (0.14)	0.45 (0.23–0.68)	<0.001 *	-
Calcium	T0	2.31 (0.017)	Ref.	Ref.	0.039
	T1	2.32 (0.019)	0.014 (−0.024–0.051)	0.466	-
	T2	2.36 (0.021)	0.053 (0.012–0.093)	0.012	-
Magnesium	T0	0.87 (0.013)	Ref.	Ref.	0.669
	T1	0.86 (0.014)	−0.009 (−0.031–0.012)	0.394	-
	T2	0.86 (0.015)	−0.007 (−0.031–0.017)	0.545	-
PTH	T0	7.0 (0.52)	Ref.	Ref.	<0.001 *
	T1	5.2 (0.56)	−1.8 (−2.5–−1.0)	<0.001 *	-
	T2	5.0 (0.58)	−2.0 (−2.8–−1.1)	<0.001 *	-
**Micronutrient**		**Geometric mean (95%CI)**	**Ratio of geometric means**	***p*** **-value of ratio**	***p*** **-value overall time**
Vitamin B6	T0	103.9 (83.9–128.6)	Ref.	Ref.	0.261
	T1	119.3 (103.0–138.2)	1.15	0.322	-
	T2	129.2 (99.7–167.5)	1.24	0.112	-
Folate	T0	20.5 (16.9–25.0)	Ref.	Ref	<0.001 *
	T1	42.5 (38.1–47.3)	2.07	<0.001 *	-
	T2	45.8 (38.5–54.6)	2.23	<0.001 *	-
Vitamin B12	T0	345.9 (268.2–446.1)	Ref.	Ref.	<0.001 *
	T1	577.3 (490.7–679.1)	1.67	0.002 *	-
	T2	603.6 (474.9–767.3)	1.75	0.001 *	-
Vitamin D	T0	47.8 (40.5–56.4)	Ref.	Ref.	<0.001 *
	T1	111.7 (98.3–126.9)	2.34	<0.001 *	-
	T2	118.3 (103.0–135.8)	2.47	<0.001 *	-
Vitamin E	T0	30.9 (29.0–32.9)	Ref.	Ref.	0.759
	T1	31.5 (27.6–35.9)	1.01	0.465	-
	T2	30.7 (25.9–36.4)	0.99	0.825	-
Ferritin	T0	41.5 (28.7–60.0)	Ref.	Ref.	<0.001 *
	T1	79.0 (54.7–114.1)	1.90	<0.001 *	-
	T2	92.6 (60.5–141.9)	2.23	<0.001 *	-
Iron	T0	11.3 (9.1–14.0)	Ref.	Ref.	<0.001 *
	T1	14.8 (12.5–17.1)	1.31	0.008 *	-
	T2	17.7 (15.1–20.6)	1.57	<0.001 *	-
Zinc Only AUMC and ZMC	T0	10.2 (8.8–12.0)	Ref.	Ref.	<0.001 *
	T1	11.9 (9.6–14.8)	1.17	0.010 *	-
	T2	13.6 (10.4–17.6)	1.33	<0.001 *	-

* Statistically significant at α = 0.01. T0 = baseline, T1 = six months after supplementation, T2 = twelve months after supplementation. AUMC = Amsterdam University Medical Center, PTH = parathyroid hormone, ZMC = Zuyderland Medical Center.

**Table 6:** Micronutrient concentrations over time for the esophagectomy group.

Micronutrient	Follow-Up Moment	Linear Mixed Model			
		Estimated Marginal Mean (±SE)	Mean Difference (95%CI)	*p*-Value	*p*-Value Overall Time
Vitamin A	T0	2.1 (0.07)	Ref.	Ref.	0.005 *
	T1	2.3 (0.081)	0.19 (0.02–0.35)	0.026	-
	T2	2.4 (0.091)	0.30 (0.12–0.48)	0.002 *	-
Vitamin E	T0	33.8 (1.0)	Ref.	Ref.	0.043
	T1	36.6 (1.1)	2.8 (0.5–5.1)	0.017	-
	T2	34.4 (1.3)	0.6 (−1.9–3.2)	0.639	-
Calcium	T0	2.35 (0.010)	Ref.	Ref.	0.006 *
	T1	2.37 (0.013)	0.017 (−0.012–0.045)	0.247	-
	T2	2.40 (0.014)	0.055 (0.025–0.085)	<0.001 *	-
Magnesium	T0	0.85 (0.008)	Ref.	Ref.	0.104
	T1	0.83 (0.009)	−0.020 (−0.038–−0.001)	0.039	**-**
	T2	0.85 (0.010)	−0.004 (−0.024–0.016)	0.707	-
Iron	T0	14.3 (0.7)	Ref.	Ref.	0.002 *
	T1	17.0 (0.8)	2.8 (1.0–4.5)	0.002 *	-
	T2	16.9 (0.9)	2.7 (0.8–4.5)	0.005 *	-
**Log-transformed model**					
**Micronutrient**	**Follow-up moment**	**Geometric mean (95%CI)**	**Ratio of geometric means**	***p*** **-value from LMM**	***p*** **-value overall time**
Vitamin B1	T0	157.3 (148.8–166.2)	Ref.	Ref.	<0.001 *
	T1	196.0 (185.9–206.7)	1.25	<0.001 *	-
	T2	195.3 (184.3–207.0)	1.24	<0.001 *	-
Vitamin B6	T0	98.1 (87.9–109.4)	Ref.	Ref.	0.005 *
	T1	111.1 (101.8–121.3)	1.13	0.039	-
	T2	121.9 (109.0–136.3)	1.24	0.001 *	-
Folate	T0	18.5 (16.4–21.0)	Ref.	Ref.	<0.001 *
	T1	43.2 (39.3–47.4)	2.34	<0.001 *	-
	T2	49.5 (44.8–54.6)	2.68	<0.001 *	-
Vitamin B12	T0	315.1 (282.0- 352.0)	Ref.	Ref.	<0.001 *
	T1	454.7 (411.9–501.8)	1.44	<0.001 *	-
	T2	500.4 (447.1–560.0)	1.59	<0.001 *	-
Vitamin D	T0	57.2 (52.8–62.1)	Ref.	Ref.	<0.001 *
	T1	119.1 (110.7–128.2)	2.08	<0.001 *	-
	T2	128.2 (118.1–139.1)	2.24	<0.001 *	-
Ferritin	T0	91.7 (74.1–113.4)	Ref.	Ref.	0.087
	T1	100.7 (84.6–119.8)	1.10	0.112	-
	T2	108.5 (89.1–132.2)	1.18	0.039	-
Zinc (Only AUMC and ZMC)	T0	13.3 (11.1–15.9)	Ref.	Ref.	<0.001 *
	T1	13.1 (10.8–16.0)	0.98	0.025	-
	T2	11.4 (10.8–12.0)	0.86	<0.001 *	-
PTH	T0	5.1 (4.4–5.7)	Ref.	Ref.	0.714
	T1	4.8 (4.2–5.4)	0.94	0.413	-
	T2	5.0 (4.1–6.1)	0.98	0.752	-

* Statistically significant at α = 0.01. T0 = baseline, T1 = six months after supplementation, T2 = twelve months after supplementation. AUMC = Amsterdam University Medical Center, PTH = Parathyroid hormone, ZMC = Zuyderland Medical Center.

## Data Availability

The datasets presented in this article are not readily available due to privacy restrictions of the subjects.

## Supplementary Materials

The following supporting information can be downloaded at: https://www.mdpi.com/article/10.3390/nu18183081/s1, Table S1: Micronutrient reference values per center; Table S2: Product composition of the multivitamin supplements; Table S3: Sensitivity analysis gastrectomy group, T0 compared to T1; Table S4: Sensitivity analysis gastrectomy group, T1 compared to T2; Table S5 Sensitivity analysis esophagectomy group, T0 compared to T1; Table S6: Sensitivity analysis esophagectomy group, T1 compared to T2; Table S7: Micronutrient deficiencies stratified for supplementation prior inclusion in the gastrectomy group; Table S8: Micronutrient deficiencies stratified for supplementation prior inclusion in the esophagectomy group; Table S9: Complete-case analysis from T0 to T2 for the gastrectomy group; Table S10: Complete-case analysis from T0 to T2 for the esophagectomy group.
