# Supplementary material for: Vitamin Insufficiency After Esophagogastric Surgery and Targeted Supplementation: A Multicenter Prospective Intervention Study Named the VITAMIN Study
Source: Nutrients. 2026 Sep 20;18(18):3081. doi: 10.3390/nu18183081 (PMC13610072; doi:10.3390/nu18183081)
Supplement: Supplementary file 1 [file nutrients-18-03081-s001.zip › nutrients-4491059-supplementary.pdf]

Supplementary

Table S1: Micronutrient reference values per center

Micronutrient	***Reference value***	***Unit***
Zuyderland Medical Center		
Vitamin A	1.0-2.8	µmol/L
Vitamin B1	100-230	nmol/L
Vitamin B6	50-200	nmol/L
Vitamin B12 total	145-569	pmol/L
Folate	8.8-60.8	nmol/L
Vitamin D	>50	
Vitamin E	12.8-41.8	µmol/L
Iron	11-30	µmol/L
Calcium	2.20-2.60	mmol/L
Ferritin	30-400	ug/l
Magnesium	0.70-1.00	mmol/L
Phosphate	0.80-1.50	mmol/L
PTH	1.7-9.2	pmol/L
Zinc	9.2-18.4	umol/l
Erasmus Medical Center		
Vitamin A	1.0–4.2	µmol/L
Vitamin B1	70–140 nmol/L	nmol/L
Vitamin B6	35–110 nmol/L	nmol/L
Vitamin B12	145–637	pmol/L
Folate	5–40	nmol/L
Vitamine D	>50	nmol/L
Vitamin E	18.5-50.1	umol/L
Iron	10-30	umol/L
Calcium	2.20-2.65	mmol/L
Ferritin male<br>Ferritin female	30–240<br>10–140	µg/L<br>µg/L
Magnesium	0.70–1.05	mmol/L
Phosphate<br>Male 60-69 yr<br>Female 60-69 yr<br>Male 70-79 yr<br>Female 70-79 yr<br>Male >80 yr<br>Female >80 yr	-<br>.0.75-1.30<br>0.88-1.46<br>0.75-1.29<br>0.88-1.45<br>0.77-1.26<br>0.87-1.43	-<br>mmol/L<br>mmol/L<br>mmol/L<br>mmol/L<br>mmol/L<br>mmol/L
Amsterdam Medical Center		

Vitamin A	1.2-2.7	µmol/L
Vitamin B1	85-200	µmol/L
Vitamin B6	35-200	nmol/L
Vitamin B12	150-670	pmol/L
Folate	5.2-34.8	nmol/L
Vitamin D	50-250	nmol/L
Vitamin E	15-35	µmol/L
Iron	-	-
Male 19-50 yr	8-39	µmol/L
Female 19-50 yr	7-35	µmol/L
Male 51-65 yr	8-35	µmol/L
Female 51-65 yr	8-30	µmol/L
Male 66-80 yr	7-31	µmol/L
Female 66-80 yr	8-30	µmol/L
Male >81 yr	8-29	µmol/L
Female >81 yr	7-28	µmol/L
Calcium	2.20-2.60	mmol/L
Ferritin	-	-
Female 30 days-50 yrs	20-150	µg/L
Female >50 yr	20-250	µg/L
Ferritin male >30 days	25-250	µg/L
Magnesium	0.71-0.98	mmol/L
Phosphate	-	-
Male >19 yr	0.62-1.32	mmol/L
Female >19 yr	0.73-1.40	mmol/L
PTH	<10	pmol/L
Zinc	10–18	µmol/l

Table S2: Product composition of the multivitamin supplements

Product composition (Multi-G)			***Product composition (Multi-E)***		
**Active ingredient-Vitamins**	**Dosage**	**RDI**	**Active ingredient-Vitamins**	**Dosage**	**RDI**
Vitamin A	1200 μg	150%	*Vitamin A*	1200 μg	150%
Vitamin B1	4,4 mg	400%	*Vitamin B1*	3,3 mg	300%
Vitamin B2	2,8 mg	200%	*Vitamin B2*	2,8 mg	200%
Niacin (vitamin B3)	32 mg	200%	*Niacin (vitamin B3)*	24 mg	150%
Vitamin B5	18 mg	300%	*Vitamin B5*	9 mg	150%
Vitamin B6	1,4 mg	100%	*Vitamin B6*	1,4 mg	100%
Biotin (vitamin B8)	100 μg	200%	*Biotin (vitamin B8)*	100 μg	200%
Folic acid (vitamin B11/B9)	700 μg	350%	*Folic acid (vitamin B11/B9)*	600 μg	300%
Vitamin B12	1000 μg	40000%	*Vitamin B12*	200 μg	8000%
Vitamin C	80mg	100%	*Vitamin C*	80 mg	100%
Vitamin D3	75 μg (3000IU)	1500%	*Vitamin D3*	75 μg (3000IU)	1500%
Vitamin E	30 mg	250%	*Vitamin E*	24 mg	200%
Vitamin K1	150 μg	200%	*Vitamin K1*	75 μg	100%
**Active ingredient-Minerals**			**Active ingredient - Minerals**		
Chromium	160 μg	400%	*Chromium*	40 μg	100%
Iron	75 mg	535%	*Iron*	32 mg	229%
Iodine	150 μg	100%	*Iodine*	150 μg	100%
Copper	3 mg	300%	*Copper*	2 mg	200%
Manganese	2 mg	100%	*Manganese*	2 mg	100%
Molybdenum	100 μg	200%	*Molybdenum*	50 μg	100%
Selenium	82,5 μg	150%	*Selenium*	55 μg	100%
Zinc	25 mg	250%	*Zinc*	16 mg	160%

Legend table 1: Multi- E will be given to patients who underwent esophagectomy and Multi-G to patients who underwent (sub)total gastrectomy. Vitamin and mineral compositions and dosage are shown of both multivitamin supplements. Both groups will receive the multivitamin supplement daily. RDI= reference intake. *IU= International Units, RDI= Reference Daily Intake.*

Tabl S3: Sensitivity analysis gastrectomy group, T0 compared to T1

**Baseline characteristics**	**Reached T1**	**Did not reach T1**	**p-value**
**Gastrectomy**	**(n=22)**	**(n=9)**	

Male (n, %)	16/22 (72.7)	5/9 (55.6)	0.417
Age [IQR]	67.5 [62.0-72.3]	74 [54.0-78.5]	0.281
BMI in kg/m² [IQR]	22.5 [20.0-24.0]	22.0 [19.0-25.5]	0.671
Smoking (n,%)	2/22 (9.1)	1/9 (11.1)	1.000
Diabetes Mellitus	1/22 (4.5)	0/9 (0.0)	1.000
Use of vitamin supplements	11/20 (55.0)	5/9 (55.6)	1.000
Vitamin B12 injections *	11/21 (52.4)	3/9 (33.3)	0.440
ASA-score (n, %)	n=22	n=9	0.170
1	3 (13.6)	4 (44.4)	
2	15 (68.2)	5 (55.6)	
3	4 (18.2)	0 (0.0)	
Median time in weeks after surgery [IQR]	27.5[19.6-116.5][	58.6.0 [9.9-219.1]	0.794
ypTNM staging (n, %)			
ypT-stage	n=22	n=9	0.608
ypT0	2 (9.1)	0 (0.0)	
ypT1a	1 (4.5)	0 (0.0)	
ypT1b	4 (18.2)	1 (11.1)	
ypT2	5 (22.7)	1 (11.1)	
ypT3	9 (40.9)	6 (66.7)	
ypN-stage	n=22	n=9	0.677
ypN0	14 (63.6)	4 (44.4)	
ypN1	5 (22.7)	3 (33.3)	
ypN2	2 (9.1)	1 (11.1)	
ypN3	1 (4.5)	1 (11.1)	

Table S4: Sensitivity analysis gastrectomy group, T1 compared to T2

**Baseline characteristics**	**Reached T2 (n=17)**	**Did not reach T2 (n=5)**	**p-value**
Male (n, %)	11/17 (64.7)	5/5 (100.0)	0.266
Age [IQR]	65 [57.0-70.5]	75 [70.5-79.0]	0.004*
BMI in kg/m^2 [IQR]	22.0 [20.0-24.0]	23.0 [20.0-28.8]	1.000
Smoking (n,%)	1/17 (5.9)	1/5 (20.0)	0.411
Diabetes Mellitus	0/17 (0.0)	1/5 (20.0)	0.227
Use of vitamin supplements	9/15 (60.0)	2/5 (40.0)	0.617
Vitamin B12 injections *	10/16 (62.5)	1/5 (20.0)	0.149
ASA-score (n, %)	n=17	n=5	1.000
1	3 (17.6)	0 (0.0)	
2	11 (64.7)	4 (80.0)	
3	3 (17.6)	1 (20.0)	
Median time in weeks after surgery [IQR]	26.3 [19.6-128.8]	30.1 [19.1-169.9]	0.845
ypTNM staging (n, %)			
ypT-stage	n=17	n=5	0.522
ypT0	2 (11.8)	0 (0.0)	
ypT1a	1 (5.9)	0 (0.0)	
ypT1b	2 (11.8)	2 (40.0)	
ypT2	5 (29.4)	0 (0.0)	
ypT3	6 (35.3)	3 (60.0)	

ypN-stage	n=17	n=5	0.533
ypN0	10 (58.8)	4 (80.0)	
ypN1	5 (29.4)	0 (0.0)	
ypN2	1 (5.9)	1 (20.0)	
ypN3	1 (5.9)	0 (0.0)	

Table S5 Sensitivity analysis esophagectomy group, T0 compared to T1

**Baseline characteristics**	**Reached T1 (n=56)**	**Did not reach T1 (n=20)**	**p-value**
Male (n, %)	47/56 (83.9)	18/20 (90.0)	0.717
Age [IQR]	68.0 [61.0-72.8]	70.1 [63.0-76.8]	0.106
BMI in kg/m^2 [IQR]	24.0 [22.0-27.0]	24.5 [22.0-27.0]	0.921
Smoking (n,%)	5/56 (8.9)	0/20 (0.0)	0.317
Diabetes Mellitus	3/56 (5.4)	2/20 (10.0)	0.602
Use of vitamin supplements	17/51 (33.3)	7/18 (38.9)	0.775
Vitamin B12 injections *	3/55 (5.5)	1/20 (5.0)	1.000
ASA-score (n, %)	n=55	n=20	0.800
1	13 (23.6)	4 (20.0)	
2	34 (61.8)	14 (70.0)	
3	8 (14.5)	2 (10.0)	
Median time in weeks after surgery [IQR]	36.6 [14.4-103.2]	46.9 [14/6-109.6]	0.645
ypTNM staging (n, %)			
ypT-stage	n=56	n=20	0.477

ypT0	8 (14.3)	1 (5.0)	
ypT1a	4 (7.1)	0 (0.0)	
ypT1b	6 (10.7)	4 (20.0)	
ypT2	10 (17.9)	5 (25.0)	
ypT3	20 (35.7)	9 (45.0)	
ypTx	8 (14.3)	1 (5.0)	
ypN-stage	n=56	n=20	0.477
ypN0	38 (67.9)	11 (55.0)	
ypN1	14 (25.0)	4 (20.0)	
ypN2	3 (5.4)	4 (20.0)	
ypN3	1 (1.8)	1 (5.0)	

Table S6: Sensitivity analysis esophagectomy group, T1 compared to T2

**Baseline characteristics**	**Reached T2 (n=43)**	**Did not reach T2 (n=13)**	**P-value**
Male (n, %)	37 (86.0)	10 (76.9)	0.419
Age [IQR]	69.0 [62.5-73.0]	59.0 [55.0-70.0]	0.079
BMI in kg/m^2 [IQR]	24.0 [22.0-27.0]	24.0 [22.0-28.0]	0.545
Smoking (n,%)	5/43 (11.6)	0/13 (0.0)	0.580
Diabetes Mellitus	2 (4.7)	1 (7.7)	0.555
Use of vitamin supplements	14/39 (35.9)	3/12 (25.0)	0.728
Vitamin B12 injections *	2/42 (4.8)	1/13 (7.7)	0.562
ASA-score (n, %)	n=42	n=13	0.026*
1	8 (19.0)	5 (38.5)	

2	30 (71.4)	4 (30.8)	
3	4 (9.5)	4 (30.8)	
Median time in weeks after surgery [IQR]	33.1 [14.7–107.0]	45.7 [19.9–99.6]	0.900
ypTNM staging (n, %)			
ypT-stage	n=43	n=13	0.731
ypT0	6 (14.0)	2 (15.4)	
ypT1a	2 (4.7)	2 (15.4)	
ypT1b	5 (11.6)	1 (7.7)	
ypT2	9 (21.4)	1 (7.7)	
ypT3	15 (34.9)	5 (38.5)	
ypTx	6 (14.0)	2 (15.4)	
ypN-stage			0.065
ypN0	30 (69.8)	8 (61.5)	
ypN1	12 (27.9)	2 (15.4)	
ypN2	1 (2.3)	2 (15.4)	
ypN3	0 (0.0)	1 (7.7)	

Table S7: Micronutrient deficiencies stratified for supplementation prior inclusion in the gastrectomy group

**Baseline deficiency**	**No prior supplementation (n,%)**	**Prior supplementation (n,%)**	***p*-value**
Vitamin B1	0/13 (0.0)	1/14 (7.1)	1.000
Vitamin B6	2/12 (16.7)	0/16 (0.0)	0.175
Folic acid	1/12 (8.3)	0/16 (0.0)	0.429
Vitamin B12	1/13 (7.7)	1/16 (6.3)	1.000
Vitamin A	0/12 (0.0)	0/15 (0.0)	-
Vitamin D	8/13 (61.5)	8/16 (50.0)	0.711
Vitamin E	0/12 (0.0)	0/15 (0.0)	-

Iron	6/13 (46.2)	6/16 (37.5)	0.716
Zinc	2/13 (15.4)	5/14 (35.7)	0.385
Ferritin	7/12 (58.3)	4/16 (25.0)	0.121
Calcium	2/13 (15.4)	2/16 (12.5)	1.000
Magnesium	0/13 (0.0)	0/16 (0.0)	-
Phosphate	0/13 (0.0)	0/16 (0.0)	-
≥1 micronutrient deficiency	11/13 (84.6)	13/16 (81.3)	1.000

Table S8: Micronutrient deficiencies stratified for supplementation prior inclusion in the esophagectomy group

**Baseline deficiency**	**No prior supplementation (n,%)**	**Prior supplementation (n,%)**	***p*-value**
Vitamin B1	1/44 (2.3)	0/22 (0.0)	1.000
Vitamin B6	1/44 (2.3)	0/21 (0.0)	1.000
Folic acid	5/44 (11.4)	0/24 (0.0)	0.153
Vitamin B12	1/44 (2.3)	1/24 (4.2)	1.000
Vitamin A	1/44 (2.3)	0/20 (0.0)	1.000
Vitamin D	20/44 (45.5)	3/24 (12.5)	0.007*
Vitamin E	0/44 (0.0)	0/20 (0.0)	-
Iron	13/45 (28.9)	7/24 (29.2)	1.000
Zinc	9/43 (20.9)	5/22 (22.7)	1.000
Ferritin	6/45 (13.3)	4/24 (16.7)	0.730
Calcium	4/44 (9.1)	1/24 (4.2)	0.649
Magnesium	1/44 (2.3)	1/24 (4.2)	1.000
Phosphate	1/44 (2.3)	0/24 (0.0)	1.000
≥1 micronutrient deficiency	34/45 (75.6)	14/24 (58.3)	0.174

***Table S9*:** Complete-case analysis from T0 to T2 for the gastrectomy group

**Micronutrient**	**Follow-up moment**	**Estimated marginal mean (±SE)**	**Mean difference (95% CI)**	***p*-value mean difference**	***p*-value overall time**
Vitamin B1 (n=14)	T0	146.3 (9.6)	Ref.	Ref.	<0.001*
	T1	195.1 (9.6)	48.8 (29.2–68.4)	<0.001	–
	T2	200.1 (9.6)	53.7 (34.1–73.4)	<0.001	–
Vitamin A (n=14)	T0	1.58 (0.14)	Ref.	Ref.	<0.001*
	T1	2.03 (0.14)	0.45 (0.20–0.69)	<0.001	–
	T2	2.08 (0.14)	0.50 (0.26–0.74)	<0.001	–

Micronutrient	Follow-up moment	Estimated marginal mean (±SE)	Mean difference (95% CI)	*p*-value mean difference	*p*-value overall time
Calcium (n=16)	T0	2.29 (0.022)	Ref.	Ref.	0.055
	T1	2.32 (0.022)	0.022 (-0.025–0.070)	0.344	–
	T2	2.35 (0.022)	0.059 (0.011–0.107)	0.018	–
Magnesium (n=16)	T0	0.864 (0.017)	Ref.	Ref.	0.971
	T1	0.864 (0.017)	-0.001 (-0.027–0.025)	0.963	–
	T2	0.861 (0.017)	-0.003 (-0.029–0.023)	0.819	–
Folic acid (n=16)	T0	24.5 (3.8)	Ref.	Ref.	<0.001*
	T1	44.2 (3.8)	19.7 (10.3–29.1)	<0.001	–
	T2	48.7 (3.8)	24.3 (14.9–33.7)	<0.001	–
Vitamin B12 (n=15)	T0	602.6 (78.5)	Ref.	Ref.	0.601
	T1	570.0 (78.5)	-32.6 (-256.0–190.9)	0.771	–
	T2	679.7 (78.5)	77.1 (-146.4–300.6)	0.491	–
Vitamin E (n=16)	T0	30.5 (2.5)	Ref.	Ref.	0.448
	T1	32.9 (2.5)	2.45 (-1.56–6.46)	0.221	–
	T2	32.2 (2.5)	1.72 (-2.29–5.73)	0.387	–
Ferritin (n=16)	T0	87.3 (28.1)	Ref.	Ref.	0.034
	T1	111.6 (28.1)	24.3 (-3.6–52.2)	0.086	–
	T2	124.4 (28.1)	37.1 (9.2–64.9)	0.011	–
Iron (n=15)	T0	13.3 (1.6)	Ref.	Ref.	0.002*
	T1	15.9 (1.6)	2.59 (-0.05–5.24)	0.054	–
	T2	18.4 (1.6)	5.04 (2.40–7.68)	<0.001	–
**Micronutrient**		**Geometric mean (95 CI)**	**Ratio of geometric means**	***p*-value of ratio**	***p*-value overall time**
PTH (n=16)	T0	6.22 (5.20–7.46)	Ref.	Ref.	<0.001*
	T1	4.48 (3.74–5.36)	0.72	<0.001	–
	T2	4.37 (3.65–5.22)	0.70	<0.001	–
Vitamin B6	T0	114.0 (87.7–148.3)	Ref.	Ref.	0.477

Micronutrient	Follow-up moment	Estimated marginal mean (±SE)	Mean difference (95% CI)	*p*-value mean difference	*p*-value overall time
(n=14)					
	T1	124.7 (95.9–161.8)	1.09	0.469	–
	T2	132.1 (101.6–171.8)	1.16	0.231	–
Vitamin D (n=15)	T0	46.0 (38.6–54.7)	Ref.	Ref.	<0.001*
	T1	113.5 (95.3–134.9)	2.47	<0.001	–
	T2	118.0 (99.3–140.6)	2.57	<0.001	–
Zinc (n=14) Only AUMC and ZMC	T0	10.8 (8.1–14.4)	Ref.	Ref.	<0.001*
	T1	12.4 (9.3–16.5)	1.15	0.013	–
	T2	13.6 (10.2–18.0)	1.26	<0.001	–

T0=baseline, T1=six months after supplementation, T2=twelve months after supplementation. AUMC=Amsterdam University Medical Center, PTH=Parathyroid hormone, ZMC=Zuyderland Medical Center.

Table S10: Complete-case analysis from T0 to T2 for the esophagectomy group

Micronutrient	Follow-up moment	Estimated marginal mean (±SE)	Mean difference (95% CI)	*p*-value mean difference	*p*-value overall time
Vitamin B1 (n=34)	T0	156.8 (6.0)	Ref.	Ref.	<0.001*
	T1	196.2 (6.0)	39.4 (27.4–51.5)	<0.001	–
	T2	200.0 (6.0)	43.2 (31.2–55.3)	<0.001	–
Vitamin A (n=31)	T0	1.97 (0.099)	Ref.	Ref.	0.004*
	T1	2.16 (0.099)	0.19 (-0.010–0.39)	0.055	–
	T2	2.30 (0.09)	0.34 (0.14–0.53)	<0.001	–
Calcium (n=34)	T0	2.34 (0.015)	Ref.	Ref.	0.006*
	T1	2.36 (0.015)	0.014 (-0.020–0.049)	0.401	–
	T2	2.40 (0.015)	0.055 (0.021–0.089)	0.002	–
Magnesium (n=35)	T0	0.850 (0.011)	Ref.	Ref.	0.237
	T1	0.835 (0.011)	-0.015 (-0.037–0.007)	0.179	–

Micronutrient	Follow-up moment	Estimated marginal mean (±SE)	Mean difference (95% CI)	*p*-value mean difference	*p*-value overall time
	T2	0.853 (0.011)	0.003 (-0.020–0.025)	0.819	–
Vitamin B6 (n=34)	T0	107.3 (9.9)	Ref.	Ref.	0.105
	T1	119.6 (9.9)	12.2 (-12.8–37.2)	0.332	–
	T2	134.3 (9.9)	27.0 (2.0–52.0)	0.034	–
Folic acid (n=37)	T0	20.7 (2.2)	Ref.	Ref.	<0.001*
	T1	46.4 (2.2)	25.7 (19.7–31.6)	<0.001	–
	T2	52.6 (2.2)	31.9 (25.9–37.9)	<0.001	–
Vitamin B12 (n=36)	T0	390.1 (33.3)	Ref.	Ref.	<0.001*
	T1	479.4 (33.3)	89.3 (28.6–150.0)	0.004	–
	T2	530.8 (33.3)	140.7 (80.0–201.4)	<0.001	–
Vitamin D (n=36)	T0	59.4 (5.3)	Ref.	Ref.	<0.001*
	T1	122.5 (5.3)	63.1 (51.9–74.3)	<0.001	–
	T2	134.3 (5.3)	74.9 (63.7–86.1)	<0.001	–
Vitamin E (n=31)	T0	33.0 (1.5)	Ref.	Ref.	0.375
	T1	35.0 (1.5)	1.98 (-0.87–4.83)	0.170	–
	T2	33.7 (1.5)	0.67 (-2.18–3.52)	0.640	–
Iron (n=37)	T0	13.0 (1.0)	Ref.	Ref.	<0.001*
	T1	16.9 (1.0)	3.92 (1.79–6.05)	<0.001	–
	T2	16.4 (1.0)	3.46 (1.33–5.59)	0.002	–
Zinc (n=31) Only AUMC and ZMC	T0	10.1 (0.28)	Ref.	Ref.	<0.001*
	T1	10.6 (0.28)	0.57 (-0.02–1.16)	0.056	–
	T2	11.5 (0.28)	1.42 (0.83–2.01)	<0.001	–
**Micronutrient**		**Geometric mean (95 CI)**	**Ratio of geometric means**	***p*-value of ratio**	***p*-value overall time**
PTH (n=37)	T0	4.98 (4.19–5.92)	Ref.	Ref.	0.971
	T1	4.84 (4.07–5.77)	0.98	0.810	–
	T2	4.90 (4.12–5.82)	0.98	0.885	–
Ferritin (n=37)	T0	87.5 (70.0–109.6)	Ref.	Ref.	0.119
	T1	100.5 (80.2–125.6)	1.15	0.154	–
	T2	106.2 (84.7–133.0)	1.21	0.045	–

T0=baseline, T1=six months after supplementation, T2=twelve months after supplementation. AUMC=Amsterdam University Medical Center, PTH=Parathyroid hormone, ZMC=Zuyderland Medical Center.
